# First report of venereal and vertical transmission of canine leishmaniosis from naturally infected dogs in Germany

**DOI:** 10.1186/1756-3305-5-67

**Published:** 2012-04-01

**Authors:** Torsten J Naucke, Susanne Lorentz

**Affiliations:** 1 Parasitus Ex e.V., Vollbergstraße 37, Niederkassel 53859, Germany; 2 Department of Zoology, Division of Parasitology, University of Hohenheim, Stuttgart 70599, Germany; 3 Laboklin GmbH & Co. KG, Steubenstraße 4, Bad Kissingen 97688, Germany

**Keywords:** Leishmaniosis, Venereal transmission, Vertical transmission, Dog, Germany

## Abstract

**Background:**

Canine leishmaniosis (CanL) is a zoonotic disease caused by *Leishmania (L.) infantum*. It is endemic to several tropical and subtropical countries but also to the Mediterranean region. It is transmitted by phlebotomine sandflies but occasional non-vector transmissions have been reported, including vertical and horizontal transmission.

**Findings:**

The authors report a case of CanL in a female boxer dog from Dusseldorf, Germany, that had never been in an endemic region. A serum sample from the bitch was tested positive for antibodies against *Leishmania *(IFAT 1:2,000, ELISA 72). The bitch had whelped three litters, and one puppy from the third litter was also found to be seropositive for *Leishmania *antibodies (IFAT 1:4,000, ELISA 78).

**Conclusions:**

Up to now, despite intensive searching, the occurrence of sandflies could not be proved in the bitch's region of origin. Thus, vertical and horizontal transmission are to be discussed as possible ways of infection. This may be the first report of venereal and vertical transmission of *L. infantum *in naturally infected dogs in Germany.

## Findings

Canine leishmaniosis (CanL) caused by *Leishmania (Leishmania) infantum *(or its New World synonym *Leishmania (L.) chagasi*) is a zoonotic disease of major public health and veterinary importance with a wide geographical distribution. It is endemic in Mediterranean countries and in regions of Africa, Asia, South and Central America [1]. But CanL is also an important concern in non-endemic regions. In Germany, increasing numbers of dogs are becoming infected by *L. infantum *as a result of travelling to Mediterranean countries, or being imported from these regions. It is estimated that there are 20,000 infected dogs in Germany [2]. Canine infection is associated with variable clinical manifestations, ranging from unapparent subclinical infections to fatal visceralizing disease. Clinical signs include generalized lymphadenomegaly, hepatomegaly, splenomegaly, fever, diarrhea, lethargy, and progressive weight loss [3,4]. Furthermore the majority of dogs show skin lesions. It is primarily a dry, exfoliative dermatitis. Further common prevalent symptoms are ulcerating skin lesions, as, for example, at the outer edge of the ear or the nose [4]. Common clinical chemistry abnormalities include hyperproteinemia observed with hypergammaglobulinemia and hypoalbuminemia [5].

**Figure 1:**
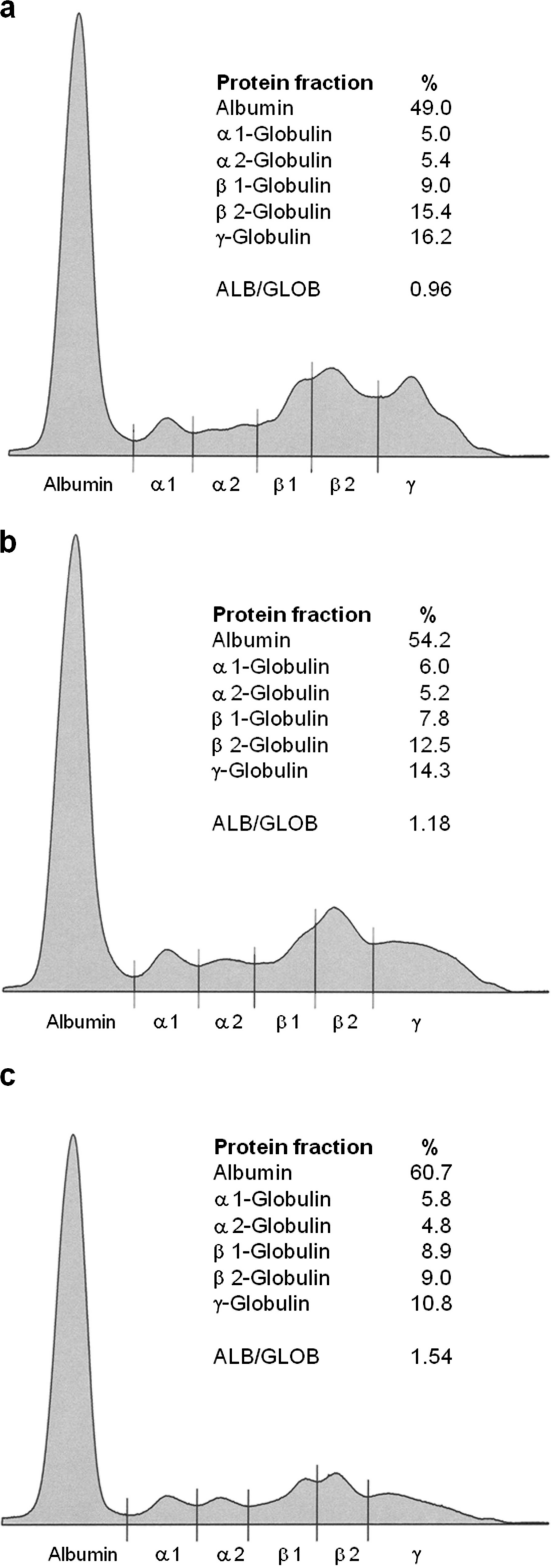
**Serum electrophoretic patterns and serum protein levels in the bitch and two puppies from the third litter**. The bitch (a) and one puppy from the third litter (b) were tested positive for antibodies against *Leishmania *(IFAT, ELISA). Serum protein electrophoresis revealed in both cases a characteristic hypergammaglobulinemia. The serum protein electrophoresis from the sample of a puppy tested negative for *Leishmania *antibodies (c) showed a gammaglobulin level within the normal range.

In spring 2011, a 7-year-old female boxer was presented in a veterinary clinic due to an exfoliative dermatitis with ulcerations on the external ears. The owners had observed that the dog became lethargic and inactive. The dog had never left the Dusseldorf region of Germany other than for a short stay in Denmark in 2005. The bitch had whelped three litters (2008 4 male, 2 female puppies; 2009 3 male puppies; 2010 2 male, 3 female puppies) from two stud dogs. In May 2011, cutaneous samples were collected at the veterinary clinic from diseased skin of the ears and of a nodular lesion at the hind limb. Histologically a lymphoplasmacytic and histiocytic inflammation, with few intracellular amastigotes in macrophages, was present. Additionally, examination of the cutaneous lesions revealed a malignant lymphoma; atypical lymphoid cells were shown to be CD3-positive in the immunohistochemical analysis. Laboratory studies revealed hyperproteinemia (89.6 g/l, reference interval 54-75 g/l), hypergammaglobulinemia (31.6%, reference interval 8-18%), hypoalbuminemia (37.1%, reference interval 47-59%), and a marginal decreased albumin/globulin-ratio (0.59, reference interval 0.59-1.11). Serologic tests, conducted in July 2011, included an enzyme-linked immunosorbent assay (ELISA, cutoff value > 5 antibody units; ELISA based on soluble promastigote antigen in combination with immunoglobuline G(γ)-specific conjugate [6]) and an indirect fluorescent antibody technique (IFAT, cutoff value > 1:50, MegaScreen^®^, MegaCor, Austria). Because IFAT sensitivity and specificity are near 100%, the test is considered by World Organization for Animal Health (OIE--Office International des Epizooties) as a reference serologic method [7]. The serum sample of the bitch was tested positive for antibodies against *Leishmania *(ELISA 72, IFAT 1:2,000). Furthermore all of the bitch's puppies were tested serologically in June 2011 and July 2011 respectively (IFAT and ELISA). One puppy of the third litter, who had never left the greater area of origin, was also found to be seropositive for *Leishmania *antibodies (ELISA 78, IFAT 1:4,000). To confirm the diagnosis of CanL in the bitch and the mentioned puppy, serum protein electrophoresis was carried out. In both cases the electrophoretic patterns revealed a hypergammaglobulinemia, a characteristic feature of CanL (Figure 1).

Based on the present investigative results, the bitch was treated with Allopurinol (20 mg/kg per day). The diagnosed T-cell lymphoma was not treated separately. The dog recovered well under the influence of the therapy and showed a good general condition ever since the beginning of the treatment.

This case of CanL demonstrates that a medical history negative for traveling to known endemic areas is insufficient to exclude CanL as a possible diagnosis. Infections with *L. infantum *in a child, as well as in a horse and dogs who had never left Germany, have already been described [8-10]. Although CanL is usually transmitted via a phlebotomine sandfly vector, alternative routes of transmission, including vertical transmission and horizontal (via direct blood to blood or sexual contact) transmission have been reported [8,11-16]. Possible modes of transmission in the reported case are discussed below.

**Figure 2:**
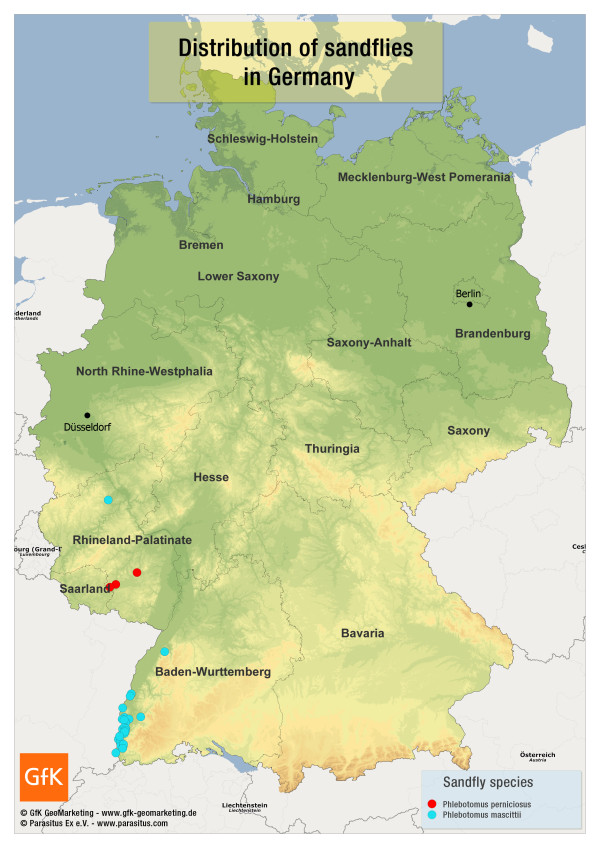
**Known geographical distribution of sandflies in Germany**. Various specimens of *Phlebotomus mascittii *were caught in different locations in Baden-Wurttemberg and one specimen near Cochem on the Mosel river. In addition, *Phlebotomus perniciosus *was detected in Germany near Kaiserslautern (Rhineland-Palatinate) [2,17].

Studies have provided evidence for the natural occurrence of sandflies in Germany. Various specimens of *Phlebotomus (P.) mascittii *were caught in different locations in Baden- Wurttemberg and one specimen near Cochem on the Mosel river. In addition, *Phlebotomus (P.) perniciosus *was detected near Kaiserslautern (Rhineland-Palatinate) [2,17] (Figure 2). Although *P. mascittii *has not yet been confirmed as a vector of leishmaniasis, its competence is suspected [2]. It is noteworthy that one specimen of *P. perniciosus*, the main vector of *L. infantum*, had been captured in 1923 in Jersey, Channel Islands, United Kingdom [18]. However, intensive searching by the authors of this short report on location since 2010 could not provide any further evidence for the natural occurrence of sandflies in that region (Naucke, unpublished observations).

Despite the installation of over one hundred sandfly traps in the greater area of Cologne/Dusseldorf/Bonn since 1999, no sandflies could be detected up to this point (Naucke, unpublished observations). This fact does not fully exclude a possible transmission of CanL via sandflies to the bitch; however, it is only with the smallest of probabilities that a sandfly transmitted the infection.

Venereal transmission has to be considered as another possible route of infection. In fact, naturally infected male dogs often develop genital lesions, particularly in the epididymis, prepuce, and glans penis and infected dogs are capable of shedding *Leishmania *in the semen [19]. In 2009, a study supported the notion that *Leishmania *may be sexually transmitted from naturally infected dogs to susceptible bitches: Twelve *Leishmania*-free bitches, housed in the absence of the insect vector, copulated with multiple infected dogs, that were shedding *Leishmania *in the semen. By the end of the experimental period, three bitches seroconverted and six were PCR positive [14]. In this reported case, both dogs that copulated with the bitch may be considered as possible vectors. The father of the first two litters died in 2010 at the age of 10 years. Stopovers in Bulgaria, Slovenia and Croatia among others for different dog shows, in addition to an artificial insemination center in Malaga, southern Spain, permit the suspicion that the dog became infected during one of these stays. Noteworthy is the especially high canine seroprevalence of CanL of 34.6% in the Axarquía region, Malaga province. In the Axarquía region, dogs belonging to short-haired breeds (boxer, setter, among others) also show a significant higher seroprevalence than those belonging to long-haired breeds [20]. The third litter's father is still alive and used for breeding. Because the CanL was only diagnosed in the bitch after the mating, a transmission of the parasite from the third litter's father to the bitch can also not be fully excluded. An inverted way of infection from the bitch to the male dogs is, from today's current knowledge, most improbable as a genital tropism of *Leishmania *has not be unequivocally proved in female dogs so far [21].

A vertical transmission of the parasite to the bitch cannot definitively be excluded, not least because the bitch herself obviously infected one of her puppies of the third litter with the parasite. The possibility of transplacental transmission of CanL is based on the circulating parasites within macrophages. The placental blood supply is in close proximity to the maternal circulation, and parasites might pass into fetal circulation [22]. Recently, two reports demonstrated vertical transmission of *L. infantum *in naturally infected dogs in a non-endemic region (North America) [11] and in an endemic region (Brazil) [13]. In North America, the vertical transmission is even discussed as a main mechanism for autochthonous cases of CanL in the foxhound population [11].

The data presented here poses new challenges and considerations for the control of CanL transmission. Disease prevention methods that solely target the vector, such as the use of repellents or avoiding travelling to endemic areas, may not be sufficient to control disease dissemination. Other methods should be considered in endemic and non-endemic regions, such as an enlargement of the breeding criteria regarding the absence of venereal or vertical transmissible infections in the dogs. That way a proven infection with CanL might be a reason for excluding dogs from professional breeding. For the future this might be especially relevant for those countries where the number of infected dogs are reaching alarming proportions, owing not only to transporting these animals to endemic areas but also due to the import of infected animals.

## Competing interests

The authors declare that they have no competing interests.

## Authors' contributions

Both authors have contributed substantially to this report. TJN collected the data and drafted the manuscript. SL carried out all laboratory examinations and helped to write the manuscript. Both authors have read and approved the final manuscript.
